# Educational Intervention Effects on Pesticide-Related Knowledge, Attitudes, Practices, Exposure, and Health Among Ugandan Smallholder Farmers: A Cluster Randomized Controlled Trial

**DOI:** 10.3389/ijph.2025.1608952

**Published:** 2025-12-03

**Authors:** Peter Ssekkadde, Vica Marie Jelena Tomberge, Curdin Brugger, Aggrey Atuhaire, Mohamed Aqiel Dalvie, Hanna-Andrea Rother, Martin Röösli, Jennifer Inauen, Mirko S. Winkler, Samuel Fuhrimann

**Affiliations:** 1 University of Basel, Basel, Switzerland; 2 Schweizerisches Tropen- und Public Health-Institut, Allschwil, Switzerland; 3 Uganda National Association of Community and Occupational Health, Kampala, Uganda; 4 University of Cape Town School of Public Health, Observatory, South Africa; 5 Universitat Bern Institut fur Psychologie, Bern, Switzerland

**Keywords:** behavior change, educational intervention, knowledge, pesticide exposure, text messages

## Abstract

**Objectives:**

This cluster randomized controlled trial assessed the effect of a two-day in-person pesticide safety training with or without text messages grounded in behavioral change theory on knowledge, attitude, and practice scores, exposure intensity scores (EIS) during application, and self-reported signs and symptoms of pesticide poisoning among 539 Ugandan smallholder farmers.

**Methods:**

Twelve subcounties were randomized into three groups: educational (n = 180), educational + text messages (n = 179), or control (n = 180). Intervention effects were estimated with mixed-effects regression models using baseline (2021) and follow-up (2022) data.

**Results:**

Knowledge scores increased by 4.4% (95% CI: 0.9, 7.8) and 6.1% (95% CI: 2.7, 9.6) in the educational and in the education + text messages groups, respectively. Attitudes increased by 6.6% (95% CI: 1.8, 11.4) with text messages. Practice scores showed no significant change. Both interventions reduced pesticide exposure, and text messages reduced signs and symptoms of pesticide poisoning by 1.1% (95% CI: −1.7, −0.3).

**Conclusion:**

The limited changes in general practices suggest that generic and content-heavy training programs may hinder implementation. Tailored behavior change approaches, identifying and addressing locally relevant practices and psychosocial drivers, may enhance farmer safety.

## Introduction

Gaps in knowledge, attitudes, and practices (KAP) along the pesticide lifecycle (e.g., mixing, application, re-entry work) can increase the risk of pesticide exposure [1]. Such gaps are common among smallholder farmers in low- and middle-income countries (LMICs), who often have limited formal education, placing them at heightened risk of occupational pesticide exposure [2, 3]. Pesticide exposure has been linked to both acute and chronic health effects [4–6]. Context-specific training or educational interventions can improve KAP and reduce exposure among smallholder farmers [7].

Interventions aimed at improving smallholder farmers’ KAP regarding pesticide handling can be categorized into four main types: (i) educational; (ii) incentive-based; (iii) technological; and (iv) regulatory, and with combined approaches proving most effective [8]. Educational interventions are widely applied across disciplines [9] and may include theoretical or practical training sessions by delivered individual facilitators or trained professionals, often incorporating guides, visual aids, or audio materials to enhance learning [9]. These interventions are typically implemented by health workers [8], extension workers or trained farmers [10] to raise awareness of pesticide risks, disseminate knowledge on safe use, and promote behavior change. Delivery formats may include lectures, workshops, group discussions [11], field visits or farmer field schools [12], and their effects can be reinforced through complementary communication strategies such as text messaging [13, 14].

In LMICs, particularly in Africa, the effective delivery of educational interventions is often constrained by multiple factors, including limited resources and a shortage of extension personnel to support large-scale implementation efforts [15]. Farmers may also encounter challenges in internalizing and applying interventions [16–18] due to complex curricula [16] or low literacy levels [2]. Furthermore, evidence on the effectiveness of existing educational interventions (e.g., training workshops), remains limited [19], which restricts opportunities for improvement and scalability. Only a few studies in Africa have evaluated the effect of educational interventions on the KAP of farmers regarding pesticide handling [10, 13, 20] using randomized controlled trials; most rely on observational designs, thereby limiting the strength of their conclusions [21].

**FIGURE 1:**
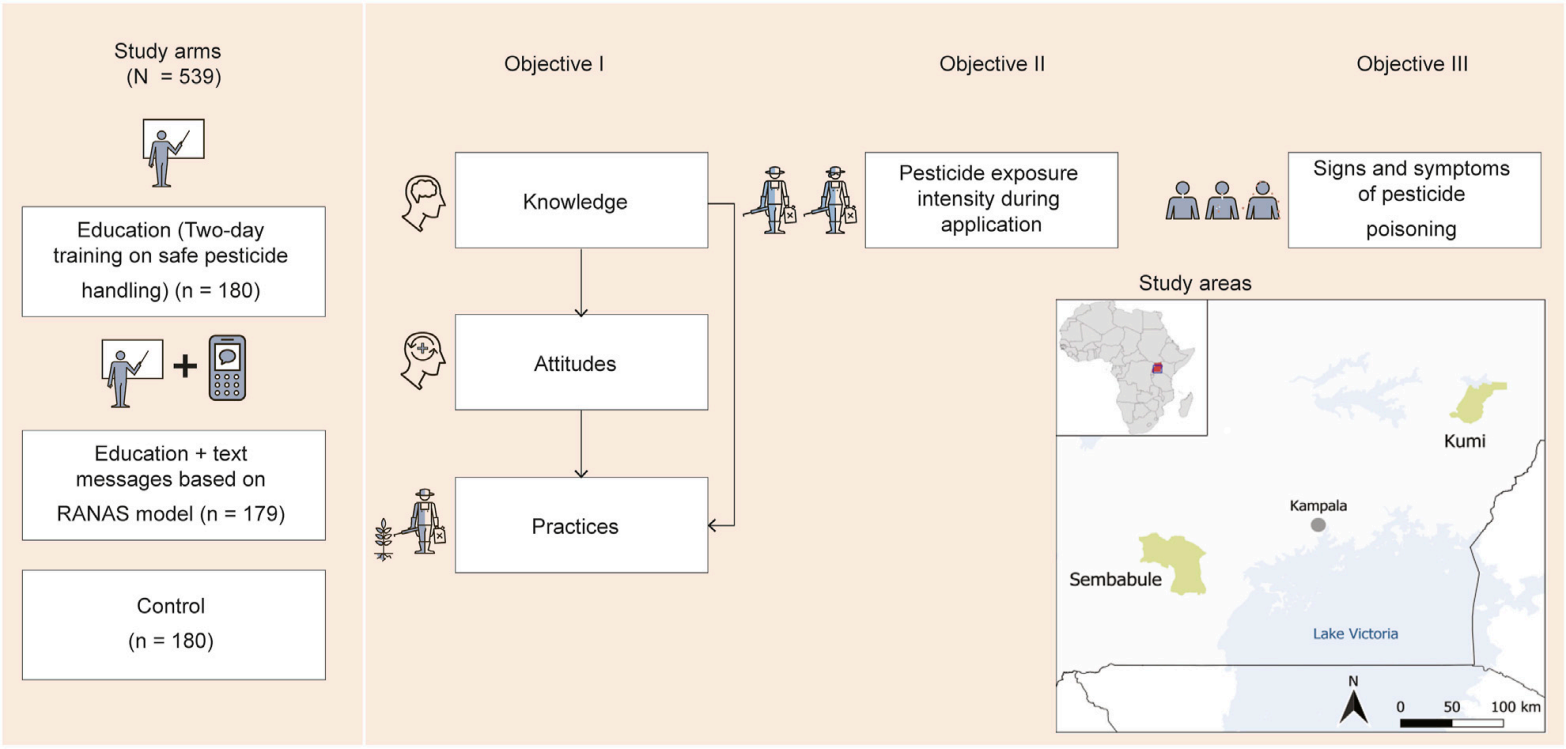
Conceptual framework of the three-arm cluster randomized trial with smallholder farmers in Uganda, showing intervention arms and assessed outcomes per study objective, and the map of Uganda indicating the study sites [Note: RANAS represents the risks, attitudes, norms, abilities, and self-regulation behavioral change approach] (African Pesticide Intervention Project, Uganda, 2020-2021).

We aimed to assess the effects of a two-day in-person educational intervention designed to improve safe pesticide handling, and a newly developed media intervention based on text messages grounded in the risks, attitudes, norms, abilities, and self-regulation (RANAS) behavior change model to improve personal protective equipment (PPE) use among smallholder farmers in Uganda. We hypothesized that the interventions would have a positive impact on outcomes. Specifically, we anticipated that the interventions would (i) improve general pesticide handling KAP scores; (ii) decrease specific pesticide exposure during application; and (iii) reduce the number of signs and symptoms of pesticide poisoning compared with the control group (Figure 1). The study was conducted as part of the African Pesticide Intervention Project (APSENT) [22].

## Methods

### Study Design and Participants

We conducted a three-arm cluster randomized controlled trial (c-RCT) with smallholder farmers in Uganda from October 2020 to October 2021. The trial was implemented across 12 subcounties (clusters) in Kumi and Sembabule districts. The 12 subcounties were randomly assigned (1:1:1) to one of the three groups using Microsoft Excel–generated random numbers by one of the investigators: (i) an educational intervention on safe pesticide handling; (ii) the educational intervention + text messages grounded in the RANAS behavior change model; and (iii) a control group that received no intervention. Structured face-to-face interviews were conducted at baseline and again 12 months later at follow-up. Participants were blinded to the group assignments of other farmers.

Farmers were eligible for inclusion in the study, if they (i) cultivated crops locally associated with high pesticide usage (watermelon, tomato, cabbage, or passion fruit), (ii) had applied pesticides within the past 12 months, (iii) were at least 18 years old, (iv) could read and write in English or the local language, (v) belonged to a household with at least one active mobile phone, and (vi) had not previously participated in training led by a local non-governmental organization (NGO), Uganda National Association for Community and Occupational Health (UNACOH).

The study is reported accordance with the CONSORT guidelines [23]. A detailed study protocol outlining the c-RCT methodology, intervention description, and farmer recruitment has been published elsewhere [22]. A short video showing the overview of the educational intervention is available at https://youtu.be/_uya5Kjay-8.

### Sample Size

We estimated a total sample size of 540 farmers, assuming 80% statistical power, based on the differences in the exposure intensity score (EIS) between trained and untrained smallholder farmers in a previous Ugandan survey [5]. The trained group had a mean EIS of 0.47 (SD = 0.10), whereas the untrained group had a mean EIS of 0.55 (SD = 0.18). The calculation was based on a two-sample means test within a cluster-randomized design comprising 12 clusters and an intraclass correlation coefficient of 0.4. A 13% dropout rate was factored into the final estimate.

### Interventions

#### Educational Intervention

The educational intervention was implemented 1 month after the baseline survey through two-day, in-person training sessions for 359 farmers. Sessions were held at a central venue in each district, with groups of approximately 45 participants. The training followed the curriculum “responsible pesticide use and handling: a guide for sustainable pest management,” developed by the local NGO, UNACOH, reviewed by the Agricultural Chemicals Board, and adopted by the Ugandan Ministry of Agriculture, Animal Industry, and Fisheries.

The curriculum comprised five modules: (i) introduction to synthetic pesticides, (ii) pesticides and human health, (iii) pesticides and the environment, (iv) pesticide application equipment, and (v) introduction to integrated pest management. Key messages for each module are presented in Supplementary Table S1. The training was delivered by the fieldwork coordinator and two local extension workers using presentations, short videos, practical demonstrations, and visual aids (e.g., pesticide labels, PPE, and pesticide application equipment). Farmer experience sharing, group exercises, and posters were also used. The training was conducted in two local languages—Ateso in Kumi and Luganda in Sembabule districts [22].

#### Text Message Intervention

Half of the farmers who attended the educational intervention in addition received 20 text messages based on the RANAS model [24]. The messages were developed using baseline data to promote the purchase and use of PPE. They were delivered in the two local languages to farmers’ mobile phones over an eight-month period following the training [22].

### Data Collection

Before data collection, trained local research assistants—with at least a bachelor’s degree and fluency in at one or more of the relevant local dialects, translated the questionnaire into three local languages and pretested it with 20 smallholder farmers [22]. Structured face-to-face interviews were conducted at participants’ homes or fields in one of the three local dialects (Luganda, Runyankore, and Ateso) or in English, using a questionnaire administered via the Open Data Kit on tablets. At the end of each day, the data was uploaded to a secure server at Swiss Tropical and Public Health Institute and reviewed for errors.

### Outcome Measurements

#### Knowledge, Attitudes and Practice (KAP) Scores

Individual scores for knowledge, attitudes, and practice scores were calculated based on 15 items per construct (range: 0–15, see Supplementary Table S2), adapted from the pesticide use in tropical settings (PESTROP) project [1, 25, 26] and aligned with four educational modules, excluding integrated pest management. Each knowledge question had a corresponding attitude and practice question [22]. Knowledge was assessed using a categorical scale (*yes* = 1, *no* = 2, and *do not know* = 3). For each correct (“yes”) response, one point was awarded while incorrect responses (“no” and “do not know”) received 0 points. Attitudes measured with a 5-point Likert scale (*strongly agree* = 1, *agree a little* = 2, *somewhat agree* = 3, *rather agree* = 4, *strongly agree* = 5). Practices were assessed with a similar scale (*never = 1, rarely = 2, sometimes = 3, often = 4, always = 5*). “Do not have an opinion” responses were coded as missing. Responses 1-3 were coded as 0 (incorrect), 4–5 as 1 (correct). Negatively worded items were reverse-scored before dichotomization. Scores were summed by domain to generate each KAP construct. For example, a perfect knowledge score corresponded to 15 correct responses.

#### Pesticide Exposure During Pesticide Application (EIS)

Pesticide exposure intensity scores (EIS; score 0–13) were assessed using a semi-quantitative, questionnaire-based algorithm tailored to the LMIC context [25, 27], as shown in Equation 1. The algorithm included five pesticide exposure modifying factors—two of which increase exposure: (i) mixing pesticides (MIX; score 5) and (ii) application using a knapsack sprayer (APPLICATION; score 8); and three reduce exposure: (i) PPE use (PPE; score 0.1–1), which accounts for access to and frequency of use of recommended PPE for six body parts (Equation 2); (ii) time to change clothes (CHANGE; score 0.7–1); and (iii) time to shower (SHOWER; score 0.7–1) after pesticide handling.

$$EIS=\left(MIX+APPLICATION\right)\;\times \;PPE\;\times \;CHANGE\;\times \;SHOWER$$ (1)

$$PPE={PPE}^{UP-BODY}+{PPE}^{MOUTH}+{PPE}^{EYE}+{PPE}^{HAND}+{PPE}^{LEG}+{PPE}^{FEET}$$ (2)

#### Signs and Symptoms of Pesticide Poisoning

Farmers self-reported experiencing one or more of 31 signs and symptoms of pesticide poisoning following pesticide application in the past 12 months (*yes* = 1, *no* = 0). These symptoms such as dizziness, nausea, and vomiting were adapted from previous studies [4, 28]. The total number of reported signs and symptoms was calculated [29] and used in subsequent analyses.

### Statistical Analysis

All statistical analyses were performed using R version 4.3.1 [30]. Continuous variables are presented as means and standard deviations and categorical variables as percentages. Intervention effects were estimated using multivariable mixed-effects models, with the follow-up outcomes as dependent variables and the study groups (control as the reference) and baseline measures as predictors. Sub-counties were included as random effects.

Sensitivity analyses included two models: (i) adjusting for age, sex, education level, income, and prior pesticide training; and (ii) combining both intervention groups to compare knowledge, attitude, and practice scores with the control group, since the text message intervention primarily targeted PPE use rather than general KAP characteristics. All analyses followed the intention-to-treat principle, with statistical significance set at *p* < 0.05. Results are reported as percentages of the maximum possible scores or values (e.g., 10/15 knowledge score = 66.7%) and regression coefficients are expressed as percentage point differences between groups.

### Deviation From the Study Protocol

Pesticide exposure was assessed without adjusting for the yearly/annual pesticide application days, as the total exceeded 365 days due to multiple products being sprayed during a single application. However, no significant group differences in yearly application days were observed at follow-up. Sensitivity analyses (ii) were conducted to assess the robustness of the findings, although these were not specified in the study protocol. Differences in outcomes between intervention recipients and nonrecipients were observed. Due to the nonconvergence in multiple imputation models, complete case data were used for the final analysis. Subanalyses were conducted on EIS subcomponents, including follow-up PPE use (PPE scores), time to change spraying clothes (CHANGE scores), and post-application showering (SHOWER scores), using study group and baseline measures as predictors.

## Results

### Baseline Demographic Characteristics of the Farmers

A total of 539 smallholder farmers participated across the three study arms: (i) educational (n = 180); educational + text messages interventions (n = 179); and control group (n = 180). Forty-five (45) farmers were lost to follow-up, resulting in 494 participants completing the study (91.7% response rate). Among the intervention groups, 30 farmers (8.4%) did not complete the two-day training, while 15 farmers (8.4%) did not receive all 20 text messages. No significant differences were observed between intervention recipients and nonrecipients, except for practice scores (odds ratio (OR) = 0.67, 95% CI: 0.46, 0.96). Participants who received the interventions were more likely to report higher practice scores (see Supplementary Table S3). Further details, including the study flowchart, are reported in the study protocol [22].

The mean age of participants was 41 years (*SD* = 12.0); 84.6% were male, and 52.3% had no formal education or only primary-level education (Table 1). Additionally, 17.6% lived below the poverty line [31] and 68.1% reported having received prior training in pesticide handling.

**TABLE 1:** Participants’ baseline social-demographic and farming characteristics across the three study groups in the randomized controlled trial with smallholder farmers in Uganda (African Pesticide Intervention Project, Uganda, 2020–2021).

Characteristics	Control (n = 180)	Education (n = 180)	Education + SMS (n = 179)
Age in years (M ± SD)	39.1 ± 11.8	44.0 ± 11.5	40.1 ± 12.3
Below 40 years (n (%))	94 (52.2)	64 (35.6)	87 (48.6)
Above 40 years (n (%))	86 (47.8)	116 (64.4)	92 (51.4)
Sex			
Male (n (%))	151 (83.9)	144 (80.0)	161 (89.9)
Female (n (%))	29 (16.1)	36 (20.0)	18 (10.1)
Education			
Primary and below (n (%))	81 (45.0)	116 (64.4)	85 (47.5)
Above primary (n (%))	99 (55.0)	64 (35.6)	94 (52.5)
Income			
Below poverty line (n (%))	29 (16.1)	35 (19.4)	31 (17.3)
Above poverty line (n (%))	151 (83.3)	145 (80.6)	148 (82.7)
Prior training in safe pesticide handling			
No (n (%))	127 (70.6)	132 (73.3)	108 (60.3)
Yes (n (%))	53 (29.4)	48 (26.7)	71 (39.7)

(1) Baseline characteristics are presented as means (M) and standard deviation (SD) for continuous variables and percentages (%) and number of participants (n) for categorical variables for each group: educational intervention (Education), education + text messages intervention (Education + SMS). (2) The poverty line is based on $1.90 per day as of October 2020 [31].

### Baseline Descriptive Summary of the Study Outcomes

**FIGURE 2:**
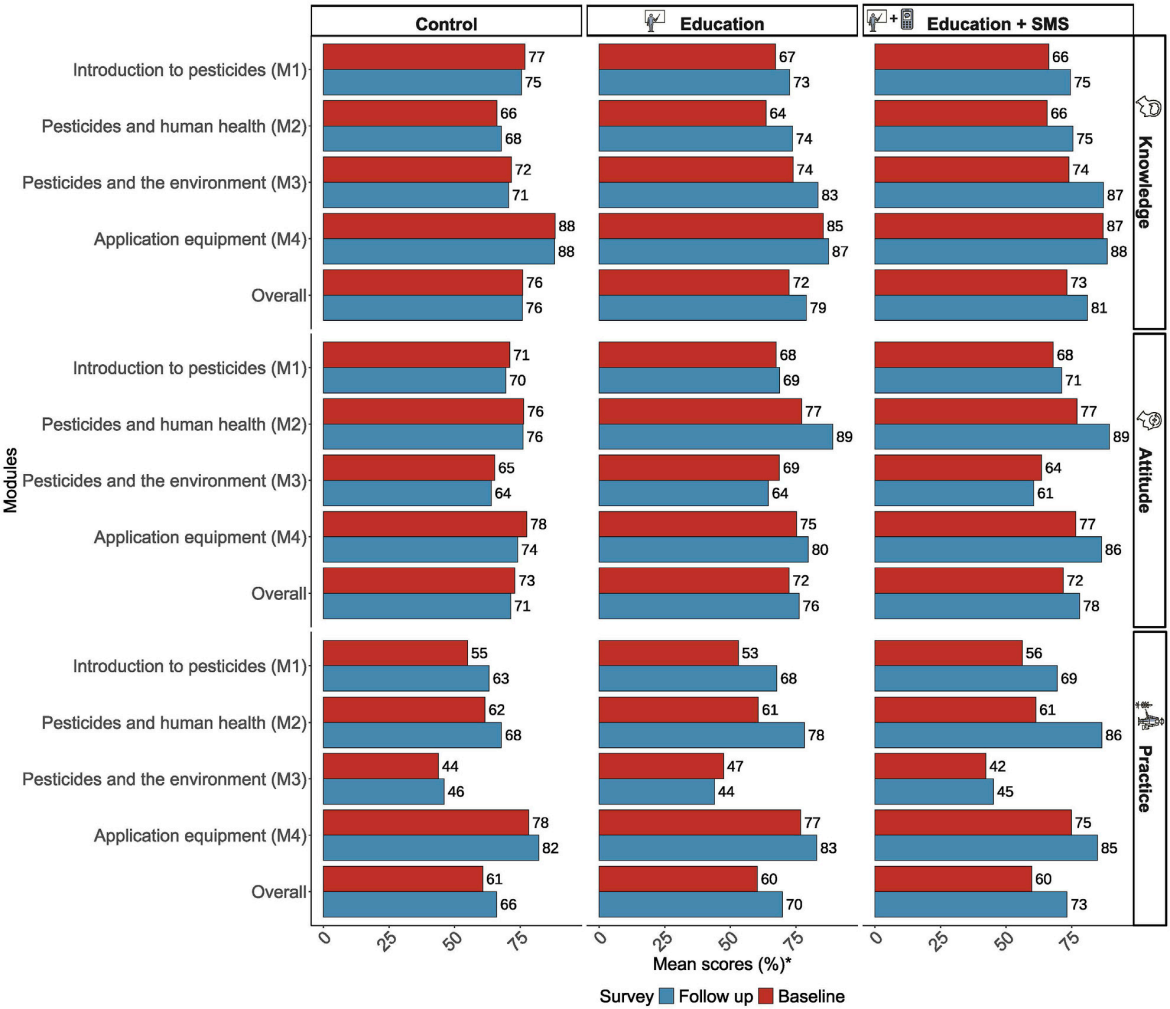
Performance of smallholder farmers in Uganda across the three study groups in a cluster randomized controlled trial, assessed based on the four modules of the safe pesticide handling curriculum [Note: Percentage mean scores* of participants in the study groups across the educational curriculum modules: introduction to pesticides (M1), pesticides and human health (M2), pesticides and the environment (M3), and common pesticide application equipment for smallholder farmers (M4)] (African Pesticide Intervention Project, Uganda, 2020-2021).

At baseline, farmers had an average knowledge score of 73.9% (SD = 11.4%), an attitude score of 72.3% (SD = 15.9%), and a practice score of 60.3% (SD = 15.8%; see Supplementary Table S4; Figure 2). The mean pesticide exposure intensity score (EIS) was 34.8% (SD = 11.5%). On average, farmers reported experiencing five (16.3% of the 31) signs or symptoms of pesticide poisoning during the 12 months preceding the interviews. Performance by individual question items and educational modules is presented in Supplementary Table S5 and Supplementary Figures S1-S3.

### Intervention Effects on Outcomes

#### KAP Scores

**FIGURE 3:**
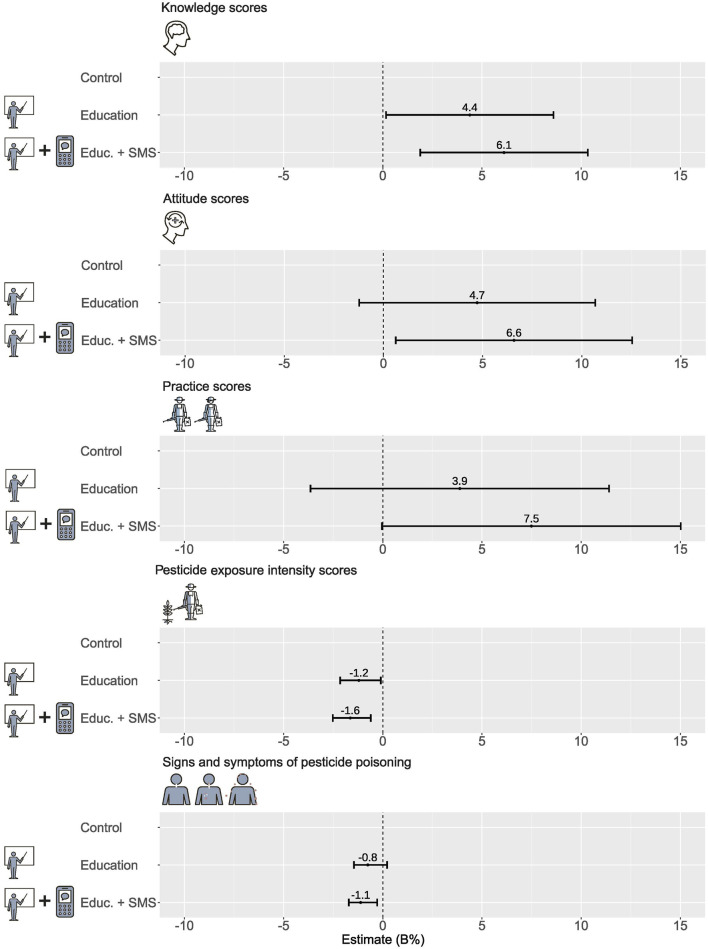
Unstandardized effect estimates of the educational and education + text message interventions on study outcomes adjusted for baseline measures and expressed as percentage proportions of the maximum possible value of each outcome [Note: B denotes unstandardized coefficient while (Educ. + SMS), the education + text message intervention] (African Pesticide Intervention Project, Uganda, 2020-2021).

At follow-up, knowledge scores increased in both the educational (B = 4.4%, 95% CI: 0.2, 8.6) and education + text message intervention (B = 6.1%, 95% CI: 1.9, 10.3) groups compared with the control group (see Figure 3; Supplementary Table S6). However, attitude scores only significantly improved in the education + text message intervention group relative to the control group (B = 6.6%, 95% CI: 0.6, 12.5). No significant changes in the practice scores were observed for either intervention group. Baseline scores were positively associated with follow-up scores of knowledge (B = 2.3%, 95% CI: 1.8, 2.8), attitudes (B = 1.4%, 95% CI: 0.9, 2.0), and practices (B = 1.5%, 95% CI: 0.9, 2.0).

A sensitivity analysis combining both intervention groups, revealed increments in knowledge (B = 5.3%, 95% CI: 1.67, 8.8) and attitude scores (B = 5.7%, 95% CI: 0.7, 10.6) compared with the control group, but not in practice scores (see Supplementary Table S7). Additional sensitivity analyses showed that farmers with education beyond primary level had higher knowledge (B = 3.3%, 95% CI: 1.5, 5.1), attitude (B = 5.6%, 95% CI: 3.3, 7.9), and practice scores (B = 3.8%, 95% CI:1.4, 6.3, 1.4) (see Supplementary Table S8). In addition, female farmers exhibited higher attitude scores than male farmers (B = 3.8%, 95% CI: 0.7, 6.8).

#### EIS During Application

At follow-up, farmers in both the educational intervention and educational + text message intervention groups showed significantly reduced EIS compared with the control group (B = −1.21%, 95% CI: −2.15, −0.10; B = −1.64%, 95% CI: −2.52, −0.61, respectively; see Supplementary Table S6). Baseline pesticide exposure scores also significantly and positively predicted the follow-up scores (B = 1.95%, 95% CI: 1.19, 2.78).

A sub-analysis of individual EIS components (see Supplementary Table S9) revealed increased PPE protection scores in both the educational (B = −11.8%, 95% CI: −19.5, −4.05), and education + text message intervention groups (B = −15.7%, 95% CI: −23.4, −8.0) compared with the control. Sensitivity analyses indicated that farmers with education beyond the primary level had significantly lower pesticide exposure scores (B = −0.4%, 95% CI: −0.8, <-0.1) than those with no formal or only primary education (Supplementary Table S8).

#### Signs and Symptoms of Acute Pesticide Poisoning

Farmers in the education + text message intervention group reported fewer signs and symptoms of pesticide poisoning during the previous 12 months compared with the control group (B = −1.1%, 95% CI: −1.7, −0.3; see Supplementary Table S6). In contrast, no significant differences in pesticide poisoning signs and symptoms were observed between the educational intervention and control groups (Supplementary Table S8), and none of the demographic characteristics predicted the health outcomes of the interventions. As with the other outcomes, baseline measures positively predicted the follow-up measures (B = 1.0%, 95% CI: 0.6, 1.3). The observed associations between the outcomes were weak (see Supplementary Table S10).

## Discussion

We conducted a three-arm c-RCT in Uganda with 539 smallholder farmers to evaluate the effects of an established two-day, in-person educational intervention on safe pesticide handling and a newly developed text message intervention grounded in the RANAS behavioral change model to promote PPE use. The interventions improved farmers’ knowledge and attitude scores. However, neither intervention resulted in significant changes in practice scores compared to the control group. Nevertheless, both intervention groups exhibited reduced EIS (i.e., exposure) during pesticide application relative to the control group. Additionally, farmers in the education + text message intervention reported fewer signs and symptoms of pesticide poisoning than those in the control group.

The observed improvements in farmers’ knowledge and attitudes toward safe pesticide handling are consistent with findings from previous studies on educational interventions [32–34], which reported positive effects on knowledge acquisition. Knowledge dissemination interventions represent a crucial initial step in promoting occupational pesticide safety by enhancing risk awareness among farming populations [1, 7, 19, 35]. Furthermore, sensitivity analysis indicated that farmers with higher levels of formal education also had higher knowledge, attitude, and practice scores. This trend could be attributed to their enhanced ability to process and retain information compared to their less educated counterparts. Prior research has identified low educational attainment as a significant barrier to effective utilization of safety knowledge, such as understanding pesticide label instructions, among Moroccan farmers [36]. Similarly, Agmas and Adugna [37] found that illiterate farmers in Ethiopia exhibited poorer attitudes towards safe pesticide use. These findings underscore the need to tailor educational interventions to low- or non-educated farming populations. Supplementing short trainings with cost-effective reminders like text messages may help sustain farmers’ general knowledge and attitudes on safe pesticide use over time.

Contrary to the observed increases in knowledge and attitudes, the interventions did not significantly improve general practices taught during the two-day training workshop (e.g., purchasing pesticides based on active ingredients, following instructions on pesticide labels and disposing pesticide containers safely). This finding aligns with a systematic review that reported limited effects of educational interventions on pesticide use practices [11]. However, reductions in pesticide exposure were observed in both intervention groups compared with the control. A parallel paper from the same study, focusing on specific practice—PPE use (not included among the 15 KAP items), revealed that both intervention groups increased farmers’ PPE use (Ssekkadde et al., unpublished results under review). The text message intervention, designed to improve PPE use by targeting locally relevant psychosocial drivers of behavioral change, led to a 16% increase in glove use at follow-up. As hands are the primary site of pesticide exposure [25], increased glove use likely contributed to the observed reductions in EIS during application and possibly to the decline in reported pesticide poisoning symptoms in the group that received text messages. Thus, although the interventions did not significantly change general practices, they improved PPE use and consequently reduced pesticide exposure.

Unlike the targeted text messages, the short training covered multiple aspects of pesticide use, potentially overwhelming the farmers with more information than they could effectively process and apply [16–18]. This information overload may have limited the training effectiveness in promoting consistent adoption of PPE use practices. Training programs that target specific aspects of pesticide handling are more likely to yield improved outcomes than broad, general interventions. For example, Mancini [38] reported reduced pesticide poisoning among Indian farmers following targeted integrated pest management training. Therefore, repeated, low-cost interventions targeting key local psychosocial factors through scalable channels such as text messages, may reinforce training benefits and improve health outcomes.

Farmers with higher baseline KAP scores demonstrated greater improvements in pesticide-related KAP at follow-up. The findings may suggest that the interventions were limited in improving outcomes beyond a certain threshold. However, farmers with higher baseline KAP scores were more likely to experience elevated pesticide exposure and associated adverse health effects. These results underscore the importance of adjusting for baseline differences when evaluating the intervention effects on key outcomes [39].

### Strengths and Limitations

Our study has three key strengths. First, we employed a c-RCT design that provides a more robust interpretation of the findings than observational studies. Second, the content of the text message intervention was developed using a rigorous RANAS behavior change approach while selecting locally relevant psychological determinants influencing particular behavior [40], such as PPE use. Third, we applied a comprehensive logical framework to evaluate the interventions, assessing their impact across KAP and specific effects on pesticide exposure and health outcomes due to pesticide application.

Our study also has three limitations worth noting: i) reliance on self-reported data may introduced reporting bias. Incorporating observational methods, such as the use of behavior checklists in data collection, could enhance the accuracy of behavioral assessments related to pesticide use; ii) future research would benefit from the inclusion of pesticide biomarkers such as cholinesterase activity in blood, to provide a more objective assessment of exposure [5]; and iii) independently evaluating the text message intervention may offer clearer insights into its effectiveness, including potential long-term effects on behavioral and health outcomes.

### Conclusion

The educational intervention improved pesticide-related knowledge, while the addition of text messages also improved farmers’ attitudes. However, both interventions had limited effects on general pesticide safety practices, likely due to the generic and content-heavy nature of traditional training programs, which can overwhelm participants and hinder practical application. Our findings show that targeting specific behaviors, such as the use of PPE within a structured behavior change framework can significantly increase adoption of these behaviors. This behavioral shift may contribute to measurable reductions in pesticide exposure and poisoning symptoms. Overall, the study highlights the importance of employing targeted behavioral approaches that identify and address locally relevant practices and psychosocial drivers. Such strategies hold promise for improving occupational safety among farmers, particularly in LMICs.
