# Supplementary material for: Educational Intervention Effects on Pesticide-Related Knowledge, Attitudes, Practices, Exposure, and Health Among Ugandan Smallholder Farmers: A Cluster Randomized Controlled Trial
Source: Int J Public Health. 2025 Dec 3;70:1608952. doi: 10.3389/ijph.2025.1608952 (PMC12708362; doi:10.3389/ijph.2025.1608952)
Supplement: Supplementary file 1 [file Supplementaryfile1.docx]

**Educational intervention effects on pesticide-related knowledge, attitudes, practices, exposure, and health among Ugandan smallholder farmers: A cluster randomized controlled trial**

**International Journal of Public Health (IJPH)**

**Table S1**

Key take-home messages per module and delivery channels used to administer the two-day in-person training (African Pesticide Intervention Project, Uganda, 2020-2021).

| Key messages per module | Delivery method (s) |
| --- | --- |
| 1. Introduction to pesticides | - PowerPoint presentation |
| - What are pesticides | - Group exercises on pesticide label interpretation |
| - How are pesticides made | - Watching short videos on pesticide modes of action, resistance development |
| - How are pesticides named (brand name vs. active ingredient) |  |
| - How are they grouped |  |
| - How pesticides work/act when applied |  |
| - How does the manufacturer communicate with the user (pesticide label interpretation) |  |
| - How do pests develop resistance to pesticides |  |
| 2. Pesticides and human health | - PowerPoint presentation |
| - Why are pesticides toxic to humans (formulation, mode of action) | - Viewing and discussing exposure photos from the field |
| - How can one identify/differentiate acute toxicity levels of different pesticides (WHO colour codes on product label) | - Group exercises on exposure and how to minimise it |
| - How do humans get exposed to pesticides: occupational, accidental, background (practices and routes of entry) | - Viewing short video clips on first aid, negative health outcomes |
| - How to different pesticides affect human health (acute and chronic effects) | - Practical demonstration with different PPEs |
| - What should one do in case of direct exposure (first aid) |  |
| - Who is vulnerable to the exposure and effects of pesticides |  |
| - How can we minimise exposure (safety precautions along the handling chain) |  |
| 3. Pesticides and the environment | - Video clips on environmental pollution from pesticides |
| - Modes of pesticide movement in the environment | - Practical demonstration of triple rinsing and puncturing of empty pesticide containers |
| - Sensitive/high-risk areas in the environment |  |
| - Effect on beneficial organisms |  |
| - Protecting the environment, e.g., Pesticide waste management |  |
| - 4. Pesticide application | - Practical demonstration of mixing and calibration exercises |
| - Pesticide application equipment (types and aspects to consider before buying a knapsack sprayer) | - Viewing different sprayers and parts |
| - Understanding different sprayer parts (especially choice of nozzles for different spray jobs), cleaning and maintenance of knapsack sprayer |  |
| - Calibration of Lever Operated Knapsack sprayer |  |
| - Mixing pesticides (Interpretation of mixing/dosage instructions on a pesticide label and right mixing procedures) |  |
| - Pesticide application techniques (to achieve precision and minimise exposure) |  |
| 5. Introduction to Integrated Pest Management (IPM) | - Practical demonstration with traps for fruit flies and fall armyworm |
| - What different approaches can be combined to manage pests and diseases | - Watching video clips |
| - How can plants resist pests or diseases, and how does this resistance develop (Host Plant Resistance) |  |
| - What ecological/cultural methods can farmers use in pest/disease management |  |
| - What are some of the beneficial insects in our fields that help to control pests (Biological control, predators, parasitoids) |  |
| - What organic pesticides/ biopesticides can farmers use, and how to access them |  |
| - Trapping of insect pests using traps (light, sticky, and pheromone traps) |  |

**Table S2**

Codebook for the knowledge, attitudes, and practice (KAP) questions of the three study groups-randomized controlled trial with smallholder farmers in Uganda at baseline and follow-up (African Pesticide Intervention Project, Uganda, 2020-2021).

| Code | Question |
| --- | --- |
| Knowledge |  |
| Label-active ingredients | The name of the pesticide active ingredients can be found on the label of the product. |
| Label-instruction | The pesticide label contains relevant information on how to handle and use the product. |
| Recommended dose | The dose recommended on the label is not a suggestion and should not be adapted by the farmer* |
| Pesticide resistance | When one pesticide is used frequently, pests get used to it and develop resistance. |
| Reuse containers | Empty pesticide containers should never be reused for packing any foodstuff at home. |
| Smelling pesticides | One does not need to smell a pesticide, to tell how toxic/hazardous it is* |
| Children’s health | Pesticides have negative effects on the health of children. |
| Non-application exposure | One can get exposed to pesticides even when not spraying* |
| Waterbody distance | Leaving a distance of at least 5 meters between your garden and a nearby waterway is a responsible practice. |
| Indiscriminate pesticides | Pesticides which kill all the insects in the field are not necessarily the most effective* |
| Normal waste | Pesticide containers should not be discharged with the normal waste* |
| Equipment leakage | Checking your spraying equipment for any leakages before mixing is a good practice. |
| Application weather | The best time to spray is not during hot/sunny times of the day* |
| Multipurpose sprayer | It is not a good practice to have one spraying equipment which can do all kinds of spraying including animals and crops* |
| Multiple-use nozzles | A good sprayer should have different types of nozzles. |
| Attitudes |  |
| Label-active ingredients | It is important for me to know the active ingredients in a given pesticide. |
| Label-instruction | It is necessary to read the instructions on the pesticide label. |
| Recommended dose | If you follow the mixing rate on the label, the pesticide works well* |
| Pesticide resistance | Pests do not only develop resistance to a pesticide if it is fake/counterfeit* |
| Reuse containers | When washed well, an empty pesticide container is still not safe to use at home* |
| Smelling pesticides | As a farmer, I am not interested in knowing the hazard level of a pesticide as long as it kills the pests on my farm. |
| Children’s health | Involving children in mixing and applying pesticides helps to equip them with farming skills at a young age. |
| Non-application exposure | I am not comfortable working in the field on the same day that I spray it* |
| Waterbody distance | When spraying, pesticide droplets drift and contaminate a nearby water point* |
| Indiscriminate pesticides | Even when there are many pests in the field, one should make the spraying mixture stronger* |
| Normal waste | It is my concern how the empty pesticide containers are discharged* |
| Equipment leakage | Testing one’s knapsack sprayer with water before mixing pesticides can save him/her from getting exposed while spraying. |
| Application weather | Personal protective clothing should only be worn whenever handling pesticides* |
| Multipurpose sprayer | Having separate spraying equipment for crops and animals is not a waste of money* |
| Multiple-use nozzles | It is necessary to have more than one nozzle type for the same spraying equipment* |
| Practices |  |
| Label-active ingredients | I look for the names of the active ingredients when buying or using a given pesticide. |
| Label-instruction | When buying and before using a new pesticide I read (or ask someone to read for me) the instructions on the label. |
| Recommended dose | I mix the recommended doses of the pesticides that I use on my crops * |
| Pesticide resistance | I change/alternate the pesticides I use based on different modes of action. |
| Reuse containers | In my home, we reuse empty pesticide containers for packing things such as sugar, salt, paraffin and others. |
| Smelling pesticides | I look at the color codes on a pesticide label to tell the hazard level of that pesticide. |
| Children’s health | In my home, I don’t allow my children to handle pesticides. |
| Non - application exposure | I do not do any other tasks in my field immediately or a few hours after spraying it with pesticides* |
| waterbody distance | I do not apply pesticides within less than 5m of nearby waterways/sources* |
| Indiscriminate pesticides | I mix strong recommended concentrations to kill all the insect pests in my field while protecting the beneficial insects. |
| Normal waste | I neither leave my empty pesticide containers in the field nor burn them. |
| Equipment leakage | I test my spraying equipment with water before the pesticide application. |
| Application weather | I mix and spray pesticides in my field independent of the weather condition. |
| Multipurpose sprayer | I do not use the same spraying equipment for my crops and animals. |
| Multiple-use nozzles | With my spraying equipment, I use different nozzles, depending on what I am spraying against |

*Note: (1) Short codes represent each full-text question for knowledge, attitude, and practice (KAP) item assessed. (2) Questions with asterisks (*) were reversed.*

**Table S3**

Differences in outcomes between receivers and non-receivers of the interventions (African Pesticide Intervention Project, Uganda, 2020-2021).

| Outcome | OR | 95% CI | | p |
| --- | --- | --- | --- | --- |
| Knowledge scores | 0.8 | 0.6 | 1.2 | 0.301 |
| Attitudes scores | 1.1 | 0.8 | 1.5 | 0.713 |
| Practice scores | 0.7 | 0.5 | 1.0 | **0.031*** |
| Exposure intensity scores | 1.0 | 0.7 | 1.4 | 0.886 |
| Signs and symptoms of  pesticide poisoning | 0.8 | 0.6 | 1.1 | 0.257 |

*Note: (1) The results are from a binomial mixed-effects regression model reporting odds ratios (ORs) comparing non-receivers to receivers at baseline, accounting for clustering at the subcounty level, 5% significance level (*p<0.05). (2) 95% CI denotes 95% confidence interval.*

**Table S4**

Baseline and follow-up statistical summary of outcome variables from the randomized controlled trial with smallholder farmers in Uganda (African Pesticide Intervention Project, Uganda, 2020-2021).

| Outcome | Overall | | | | Control | | | | Education | | | | Education + SMS | | | |
| --- | --- | --- | --- | --- | --- | --- | --- | --- | --- | --- | --- | --- | --- | --- | --- | --- |
|  | BL  (n = 539) | | FU  (n = 494) | | BL  (n =180) | | FU  (n = 162) | | BL  (n = 180) | | FU  (n = 169) | | BL  (n = 179) | | FU  (n = 163) | |
|  | M | SD | M | SD | M | SD | M | SD | M | SD | M | SD | M | SD | M | SD |
| Knowledge scores | 73.9 | 11.4 | 78.7 | 10.5 | 76.0 | 11.1 | 75.9 | 11.8 | 72.5 | 12.3 | 79.0 | 9.9 | 73.1 | 10.5 | 81.0 | 8.9 |
| Attitude scores | 72.3 | 15.9 | 75.2 | 13.3 | 73.0 | 15.5 | 71.3 | 14.3 | 72.2 | 16.9 | 76.3 | 13.0 | 71.7 | 15.1 | 77.9 | 11.6 |
| Practice scores | 60.3 | 15.8 | 69.7 | 14.3 | 60.7 | 16.5 | 66.1 | 14.4 | 60.3 | 16.1 | 69.8 | 14.7 | 59.7 | 14.7 | 73.3 | 12.9 |
| Exposure intensity scores | 34.8 | 11.5 | 31.4 | 11.1 | 33.5 | 12.5 | 36.4 | 10.9 | 35.8 | 10.9 | 30.5 | 10.9 | 35.1 | 11.1 | 27.4 | 9.4 |
| Signs and symptoms of pesticide poisoning | 16.3 | 18.4 | 10.8 | 14.7 | 18.4 | 18.4 | 14.4 | 15.4 | 13.9 | 17.4 | 10.3 | 16.4 | 16.7 | 19.1 | 7.7 | 11.2 |

*Note: (1) Participants’ baseline and follow-up outcome means and standard deviations (SD) are presented as percentage proportions (%) of the maximum possible scores of the outcomes (e.g., 10 of 15 for knowledge scores is equivalent to 66.67%). (2) n represents the overall number of participants in each study group.*

**Table S5**

Differences in overall mean scores at baseline and follow-up for individual questions per individual knowledge, attitudes, and practice domain items (African Pesticide Intervention Project, Uganda, 2020-2021).

| Item | Code | Baseline  mean score (%) | Follow-up  mean score (%) | p-value |
| --- | --- | --- | --- | --- |
| Knowledge | Label-active ingredients | 77.55 | 88.66 | **0.001*** |
|  | waterbody distance | 91.28 | 95.34 | **0.003*** |
|  | Indiscriminate pesticides | 57.14 | 68.22 | **0.001*** |
|  | Normal waste | 71.06 | 77.73 | **0.005*** |
|  | Equipment leakage | 97.77 | 98.99 | 0.071 |
|  | Application weather | 66.98 | 64.98 | 0.310 |
|  | Multipurpose sprayer | 92.76 | 95.14 | 0.158 |
|  | Multiple-use nozzles | 90.17 | 92.91 | **0.039*** |
|  | Label-instruction | 94.99 | 97.77 | **0.016*** |
|  | Recommended dose | 24.30 | 21.86 | 0.425 |
|  | Pesticide resistance | 83.49 | 88.46 | **0.016*** |
|  | Reuse containers | 89.24 | 95.75 | **0.001*** |
|  | Smelling pesticides | 17.44 | 30.57 | **0.001*** |
|  | Children’s health | 98.52 | 97.77 | 0.286 |
|  | Non - application exposure | 55.29 | 65.38 | **0.001*** |
|  |  |  |  |  |
| Attitudes | Label-active ingredients | 86.78 | 92.11 | **0.003*** |
|  | waterbody distance | 68.66 | 52.23 | **0.001*** |
|  | Indiscriminate pesticides | 40.89 | 44.53 | 0.252 |
|  | Normal waste | 87.94 | 92.31 | **0.007*** |
|  | Equipment leakage | 81.08 | 88.06 | **0.001*** |
|  | Application weather | 49.17 | 51.21 | 0.224 |
|  | Multipurpose sprayer | 91.84 | 94.74 | 0.057 |
|  | Multiple-use nozzles | 83.64 | 86.23 | 0.230 |
|  | Label-instruction | 92.95 | 95.95 | **0.024*** |
|  | Recommended dose | 63.50 | 68.83 | **0.033*** |
|  | Pesticide resistance | 32.16 | 22.31 | **0.001*** |
|  | Reuse containers | 84.42 | 91.90 | **0.001*** |
|  | Smelling pesticides | 75.65 | 80.16 | 0.059 |
|  | Children’s health | 72.36 | 86.44 | **0.001*** |
|  | Non - application exposure | 75.14 | 81.17 | **0.008*** |
|  |  |  |  |  |
| Practices | Label-active ingredients | 41.37 | 62.55 | **0.001*** |
|  | waterbody distance | 51.76 | 36.44 | **0.001*** |
|  | Indiscriminate pesticides | 53.43 | 63.97 | **0.001*** |
|  | Normal waste | 28.39 | 34.82 | **0.024*** |
|  | Equipment leakage | 77.74 | 87.25 | **0.001*** |
|  | Application weather | 66.42 | 70.99 | 0.194 |
|  | Multipurpose sprayer | 85.69 | 92.11 | **0.001*** |
|  | Multiple-use nozzles | 76.81 | 82.79 | **0.005*** |
|  | Label-instruction | 72.17 | 88.44 | **0.001*** |
|  | Recommended dose | 42.67 | 48.58 | **0.010*** |
|  | Pesticide resistance | 62.71 | 67.81 | 0.080 |
|  | Reuse containers | 95.55 | 96.76 | 0.274 |
|  | Smelling pesticides | 22.08 | 65.38 | **0.001*** |
|  | Children’s health | 62.08 | 73.89 | **0.001*** |
|  | Non - application exposure | 65.12 | 74.29 | **0.001*** |

*Note: (1) Paired t-tests were used to assess baseline and follow-up individual KAP questions percentage mean score (%) differences at 5% significance level (*p <0.05).*


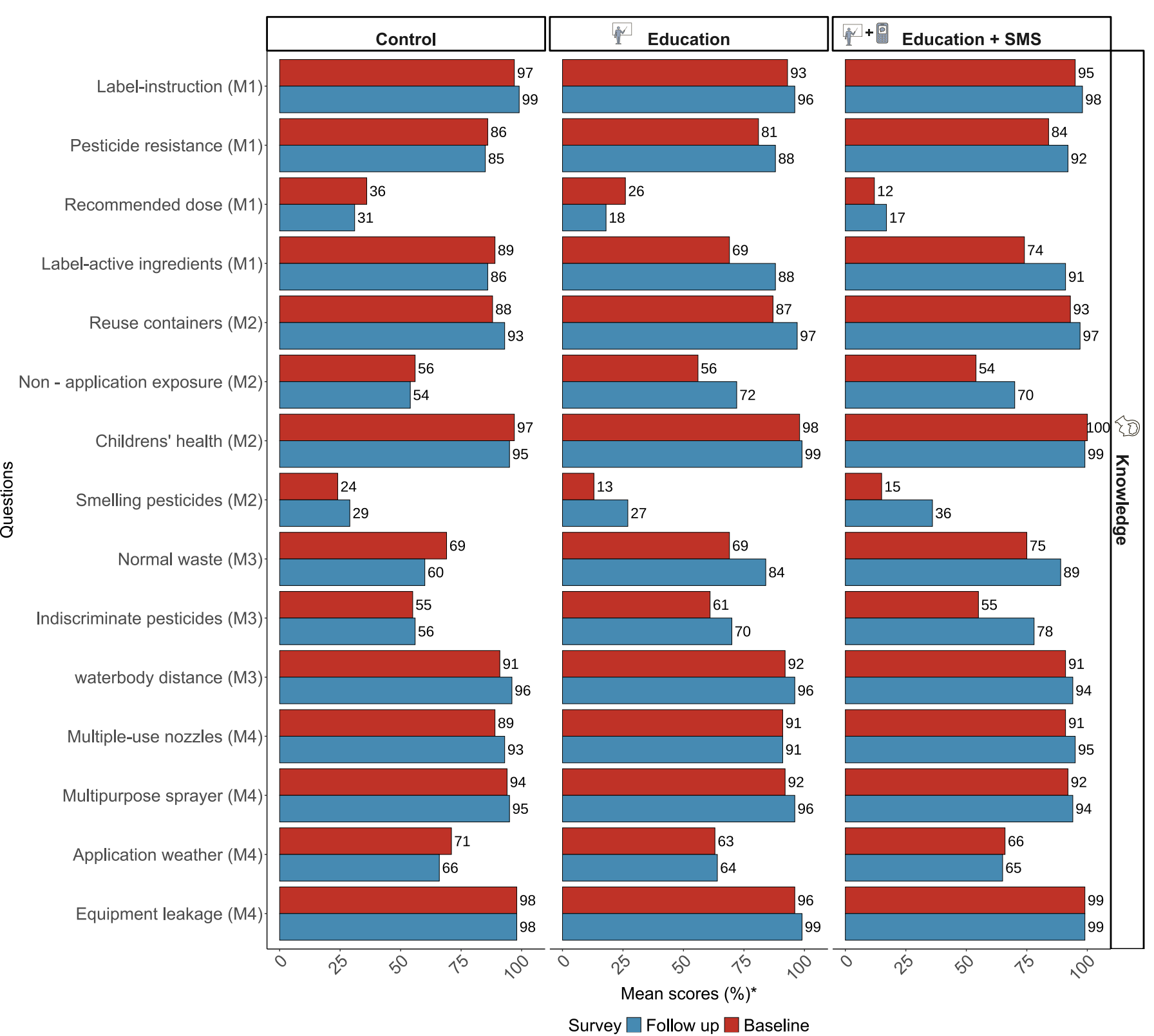


**Figure S1**. Graphical distribution of knowledge scores along the pesticide handling curriculum of the three study groups of a cluster randomized controlled trial with smallholder farmers in Uganda at baseline and follow-up [Note: (1) The specific knowledge questions’ mean scores* represented by short codes on the x-axis (full code text shown in Supplementary Table S2). (2) Responses to the questions are based on a binary scale: yes = 1, no = 2, and don’t know = 3. A response of 1(100%) is considered correct, while 2 and 3 (0%) are regarded as wrong. (3) Items corresponding to each module are indicated in brackets as follows: introduction to pesticides (M1), pesticides and human health (M2), pesticides and the environment (M3), and common pesticide application equipment for smallholder farmers (M4)] (African Pesticide Intervention Project, Uganda, 2020-2021).

**
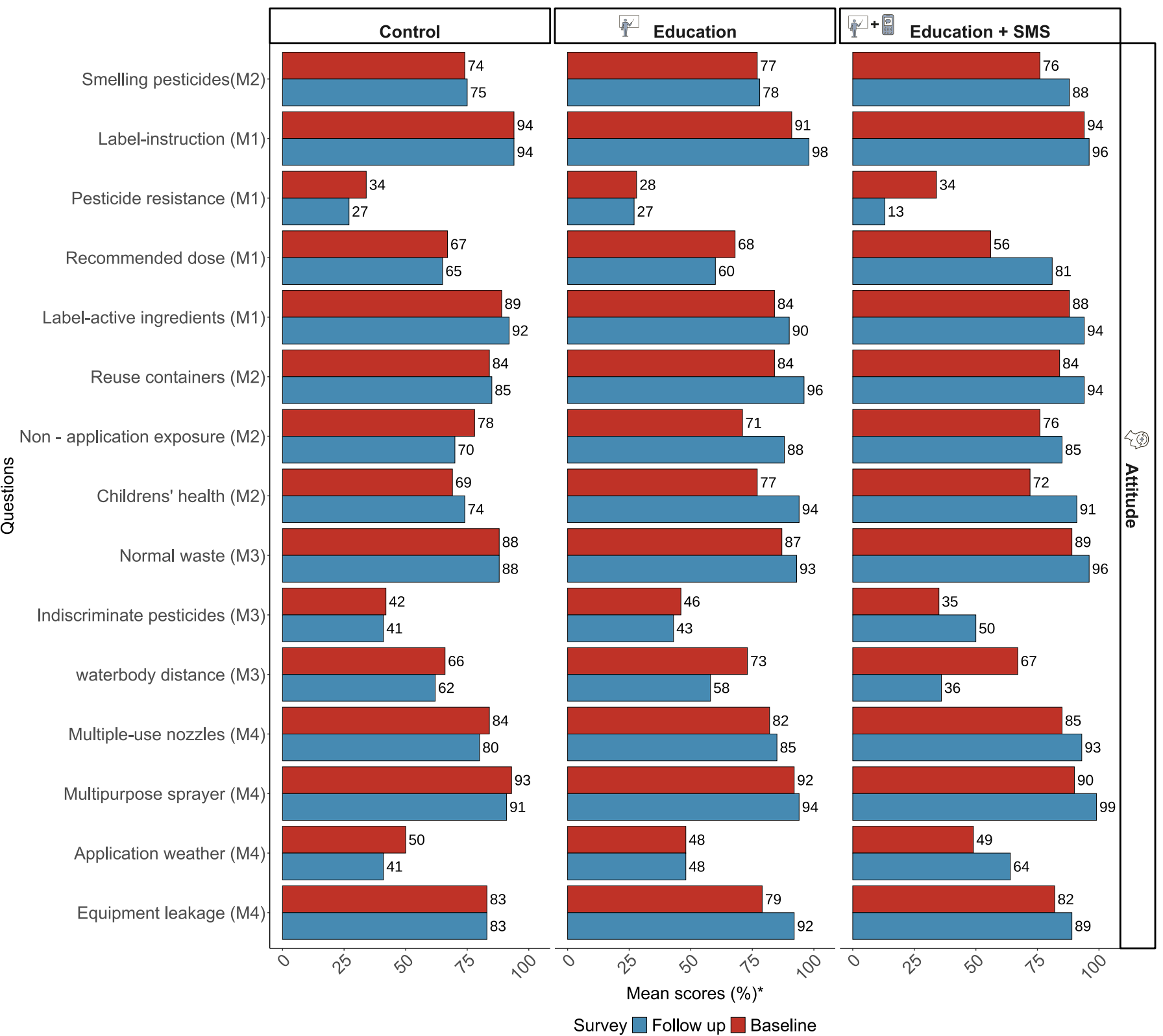
**

**Figure S2**. Graphical distribution of attitude scores along the pesticide handling curriculum of the three study groups of a randomized controlled trial with smallholder farmers in Uganda at baseline and follow-up [Note: (1) The specific attitude questions' percentage mean scores* represented by short codes on the x-axis (full code text shown in Supplementary Table S2). (2) Responses to the questions were based on a Likert scale: agree not at all to agree=1, agree a little=2, somewhat agree=3, rather agree=4, and strongly agree=5. A response of 1 to 3 (0%) is considered wrong, while 4 and 5 are regarded as correct (100%). (3) Items corresponding to each module are indicated in brackets as follows: introduction to pesticides (M1), pesticides and human health (M2), pesticides and the environment (M3), and common pesticide application equipment for smallholder farmers (M4)] (African Pesticide Intervention Project, Uganda, 2020-2021).

**
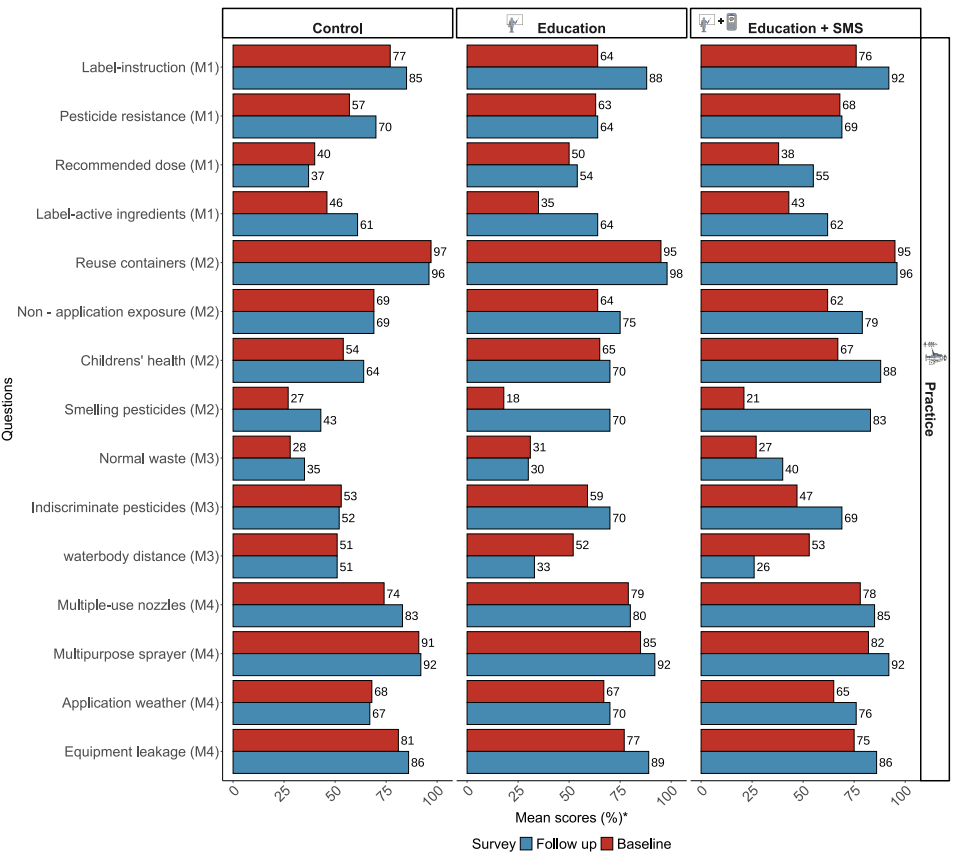
**

**Figure S3**. Graphical distribution of practice scores along the pesticide handling curriculum of the three study groups of a randomized controlled trial with smallholder farmers in Uganda at baseline and follow-up. [Note: (1) The specific practice questions’ percentage mean scores* represented by codes on the x-axis (full code text shown in Supplementary Table S2). (2) Responses to the questions are based on a Likert scale: never=1, rarely=2, sometimes=3, often=4, and always=5. A response of 1 to 3 is considered wrong (0%), while 4 and 5 are correct (100%). (3) Items corresponding to each module are indicated in brackets as follows: introduction to pesticides (M1), pesticides and human health (M2), pesticides and the environment (M3), and common pesticide application equipment for smallholder farmers (M4)] (African Pesticide Intervention Project, Uganda, 2020-2021).

**Table S6**

Multivariable analysis summary of the effect of interventions on the outcomes across the three study groups in the randomized controlled trial with smallholder farmers in Uganda (African Pesticide Intervention Project, Uganda, 2020-2021).

| Outcome | B (%) | 95% CI | | p-value |
| --- | --- | --- | --- | --- |
| Knowledge scores |  |  | |  |
| Education | 4.4 | 0.2 | 8.6 | **0.043*** |
| Education + SMS | 6.1 | 1.9 | 10.3 | **0.010*** |
| Baseline score | 2.3 | 1.8 | 2.8 | **0.001*** |
| Attitude scores |  |  |  |  |
| Education | 4.7 | -1.2 | 10.7 | 0.106 |
| Education + SMS | 6.6 | 0.6 | 12.5 | **0.034*** |
| Baseline score | 1.4 | 0.9 | 2.0 | **0.001*** |
| Practice scores |  |  |  |  |
| Education | 3.9 | -3.7 | 11.4 | 0.273 |
| Education + SMS | 7.5 | 0.0 | 15.0 | 0.051 |
| Baseline score | 1.5 | 0.9 | 2.0 | **0.001*** |
| Pesticide exposure intensity scores (EIS) |  |  |  |  |
| Education | -1.2 | -2.2 | -0.1 | **0.036*** |
| Education + SMS | -1.6 | -2.5 | -0.6 | **0.007*** |
| Baseline score | 2.0 | 1.2 | 2.8 | **0.001*** |
| Signs and symptoms of  pesticide poisoning |  |  |  |  |
| Education | -0.8 | -1.5 | 0.2 | 0.101 |
| Education + SMS | -1.1 | -1.7 | -0.3 | **0.018*** |
| Baseline measure | 1.0 | 0.6 | 1.3 | **0.001*** |

*Note: (1) Mixed effects regression model summaries of the outcomes indicate the unstandardized estimates (B) of educational (Education) and education + text message (Education + SMS) interventions with the control group as the reference group. (2) The results are expressed as percentages of the maximum possible outcome score or measure (e.g., 10 of the 15 possible maximum knowledge scores is equivalent to 66.7%). (3) *p<0.05 denotes significance at 5% while 95% CI, 95% confidence interval.*

**Table S7**

Multivariable analysis summary of effects of combined interventions (educational and educational and text interventions on knowledge, attitudes, and practices across the three study groups in the randomized controlled trial with smallholder farmers in Uganda, accounting for age, sex, education, and prior training in pesticide handling (African Pesticide Intervention Project, Uganda, 2020-2021).

| Outcome | B (%) | 95% CI | | P-value |
| --- | --- | --- | --- | --- |
| Knowledge scores |  |  |  |  |
| Education | 5.3 | 1.7 | 8.8 | **0.008*** |
| Baseline scores | 2.3 | 1.8 | 2.8 | **<.001*** |
|  |  |  |  |  |
| Attitude score |  |  |  |  |
| Education | 5.7 | 0.7 | 10.6 | **0.029*** |
| Baseline scores | 1.4 | 0.9 | 2.0 | **<.001*** |
|  |  |  |  |  |
| Practice scores |  |  |  |  |
| Education | 1.5 | 0.9 | 2.0 | 0.080 |
| Baseline score | 49.6 | 43.3 | 56.0 | **<.001*** |

*Note: (1) Mixed effects regression model summaries of the KAP outcomes indicate the unstandardized estimates (B) of educational and educational + text message interventions (combined) with the control group as the reference group. (2) The results are expressed as percentages of the maximum possible outcome score or measure (e.g., 10 of the 15 possible maximum knowledge scores is equivalent to 66.7%). (3) *p<0.05 denotes significance at 5% while 95% CI, 95% confidence interval.*

**Table S8**

Multivariable analysis summary of the effect of interventions on the outcomes across the three study groups in the randomized controlled trial with smallholder farmers in Uganda, with age, sex, education, and prior training in pesticide handling as cofounders (African Pesticide Intervention Project, Uganda, 2020-2021).

| Outcome | B (%) | 95% CI | | p-value |
| --- | --- | --- | --- | --- |
| Knowledge scores |  |  |  |  |
| Education | 5.1 | 0.6 | 9.6 | **0.031*** |
| Educ.+ SMS | 6.3 | 1.7 | 10.8 | **0.012*** |
| Baseline score | 2.0 | 1.5 | 2.6 | **0.001*** |
| Female | -1.7 | -4.1 | 0.6 | 0.150 |
| Above the poverty line | 2.1 | -0.1 | 4.2 | 0.060 |
| Above primary | 3.3 | 1.5 | 5.1 | **0.001*** |
| Above 40 years | -1.0 | -2.8 | 0.7 | 0.246 |
| Prior training in pesticide use | -1.3 | -3.1 | 0.5 | 0.157 |
| Attitude scores |  |  |  |  |
| Education | 5.4 | -0.3 | 11.1 | 0.059 |
| Educ.+ SMS | 6.8 | 1.1 | 12.5 | **0.024*** |
| Baseline score | 1.2 | 0.6 | 1.7 | **0.001*** |
| Female | 3.8 | 0.7 | 6.8 | **0.017*** |
| Above the poverty line | 2.2 | -0.6 | 5.0 | 0.130 |
| Above primary | 5.6 | 3.3 | 7.9 | **0.001*** |
| Above 40 years | 1.6 | -0.7 | 3.9 | 0.167 |
| Prior training in pesticide use | 1.1 | -1.2 | 3.4 | 0.361 |
| Practice scores |  |  |  |  |
| Education | 4.7 | -2.4 | 11.7 | 0.169 |
| Educ.+ SMS | 7.6 | 0.5 | 14.6 | **0.038*** |
| Baseline score | 1.4 | 0.8 | 1.9 | **0.001*** |
| Female | 0.7 | -2.6 | 4.0 | 0.693 |
| Above the poverty line | -1.4 | -4.5 | 1.6 | 0.355 |
| Above primary | 3.8 | 1.4 | 6.3 | **0.002*** |
| Above 40 years | -0.9 | -3.3 | 1.6 | 0.495 |
| Prior training in pesticide use | 0.8 | -1.7 | 3.3 | 0.549 |
| Pesticide exposure intensity scores (EIS) |  |  |  |  |
| Education | -1.2 | -2.1 | -0.2 | **0.029*** |
| Educ.+ SMS | -1.6 | -2.5 | -0.6 | **0.006*** |
| Baseline score | 1.9 | 1.1 | 2.8 | **0.001*** |
| Female | 0.0 | -0.5 | 0.6 | 0.887 |
| Above the poverty line | 0.0 | -0.5 | 0.5 | 0.903 |
| Above primary | -0.4 | -0.8 | 0.0 | 0.050 |
| Above 40 years | -0.4 | -0.8 | 0.0 | 0.077 |
| Prior training in pesticide use | 0.0 | -0.5 | 0.4 | 0.851 |
| Signs and symptoms of pesticide  poisoning (%) | |  |  |  |
| Education | 13.4 | 7.3 | 22.1 | 0.106 |
| Educ.+ SMS | -1.9 | -3.7 | 0.6 | **0.025*** |
| Baseline measure | -2.7 | -4.2 | -0.5 | **0.001*** |
| Female | 2.2 | 1.4 | 3.1 | 0.125 |
| Above the poverty line | -1.2 | -2.5 | 0.4 | 0.635 |
| Above primary | 0.4 | -1.1 | 2.1 | 0.060 |
| Above 40 years | -1.1 | -2.1 | 0.1 | 0.810 |
| Prior training in pesticide use | -0.2 | -1.3 | 1.2 | 0.307 |

*Note: (1) Mixed effects regression model summaries of the outcomes with covariates indicate the unstandardized estimates (B) of educational (Education) and education + text message (Education + SMS) interventions with the control group as the reference group. (2) The results are expressed as percentages of the maximum possible outcome score or measure (e.g., 10 of the 15 possible maximum knowledge scores is equivalent to 66.7%). (3) *p<0.05 denotes significance at 5%, while 95% CI, 95% confidence interval.*

**Table S9**

Multivariable analysis summary of effects of interventions on pesticide exposure modifying factors across the three study groups in the randomized controlled trial with smallholder farmers in Uganda (African Pesticide Intervention Project, Uganda, 2020-2021).

| Outcome | B (%) | 95% CI | | P-value |
| --- | --- | --- | --- | --- |
| PPE protection scores |  |  |  |  |
| Education | -11.3 | -19.0 | -3.5 | **0.009*** |
| Education + SMS | -16.1 | -23.8 | -8.3 | **0.001*** |
| Baseline score | 27.2 | 18.9 | 35.4 | **<.001*** |
| Change scores |  |  |  |  |
| Education | 0.2 | -3.6 | 4.0 | 0.895 |
| Education + SMS | -0.8 | -4.6 | 3.0 | 0.646 |
| Baseline score | 8.5 | 1.5 | 15.5 | **0.018*** |
| Shower scores |  |  |  |  |
| Education | 0.0 | -3.6 | 3.7 | 0.980 |
| Education + SMS | -0.8 | -4.4 | 2.9 | 0.658 |
| Baseline score | 11.9 | 5.2 | 18.7 | **0.001*** |

*Note: (1) Mixed effects regression model summaries of the pesticide exposure components indicate the estimates (B) of Educational (Education) and educational + text message (Education + SMS) interventions with the control group as the reference group. (2) The results are expressed as percentages of the maximum possible outcome score or measure (e.g., 0.5 of the maximum 1 score of PPE (personal protective equipment) protection is equivalent to 50%). (3) *p<0.05 denotes the significance at 5% while 95% CI, 95% confidence interval*.

**Table S10**

Correlation matrix of the baseline and follow-up outcomes of the randomized controlled trial with smallholder farmers in Uganda (African Pesticide Intervention Project, Uganda, 2020-2021).

| Outcome | EIS.BL | EIS.FU | H.BL | H.FU | K.BL | A.FU | P.BL | K.FU | A.BL | P.FU |
| --- | --- | --- | --- | --- | --- | --- | --- | --- | --- | --- |
| EIS.BL | 1.00 | 0.21* | 0.11* | 0.03 | -0.14* | -0.04 | -0.23* | -0.07 | -0.14* | -0.14* |
| EIS.FU | 0.21* | 1.00 | 0.09 | 0.20* | -0.09 | -0.04 | -0.06 | -0.08 | -0.22* | -0.30* |
| H.BL | 0.11* | 0.09 | 1.00 | 0.30* | 0.08 | 0.07 | 0.19* | -0.10* | -0.07 | -0.02 |
| H.FU | 0.03 | 0.20* | 0.30* | 1.00 | 0.02 | 0.03 | -0.01 | -0.11* | -0.19* | -0.10* |
| K.BL | -0.14* | -0.09 | 0.08 | 0.02 | 1.00 | 0.38* | 0.36* | 0.32* | 0.12* | 0.15* |
| A.FU | -0.04 | -0.04 | 0.07 | 0.03 | 0.38* | 1.00 | 0.38* | 0.16* | 0.28* | 0.32* |
| P.BL | -0.23* | -0.06 | 0.19 | -0.01 | 0.36* | 0.38* | 1.00 | 0.05 | 0.12* | 0.30* |
| K.FU | -0.07 | -0.08 | -0.10 | -0.11* | 0.32* | 0.16* | 0.05 | 1.00 | 0.27* | 0.15* |
| A.BL | -0.14* | -0.22* | -0.07 | -0.19* | 0.12* | 0.28* | 0.12* | 0.27* | 1.00 | 0.32* |
| P.FU | -0.14* | -0.30* | -0.02 | -0.10* | 0.15* | 0.32* | 0.30* | 0.15* | 0.32* | 1.00 |

*Note: (1) Spearman correlation coefficients of baseline (BL suffix) and follow-up (FL suffix) for outcomes. (2) Pesticide exposure intensity scores (EIS). (3) Number of health signs and symptoms of pesticide poisoning (H). (4) Knowledge (K), attitudes (A) and practices (P) scores. *p<0.05* *represents significance at 5% level.*
